# Heterogeneity of tertiary lymphoid structures in cancer

**DOI:** 10.3389/fimmu.2023.1286850

**Published:** 2023-12-04

**Authors:** Xin You, Kristina Koop, Andreas Weigert

**Affiliations:** ^1^ Goethe-University Frankfurt, Faculty of Medicine, Institute of Biochemistry I, Frankfurt, Germany; ^2^ First Department of Medicine, Universitätsklinikum Erlangen, Friedrich-Alexander-Universität Erlangen-Nürnberg, Erlangen, Germany; ^3^ Frankfurt Cancer Institute, Goethe-University Frankfurt, Frankfurt, Germany; ^4^ German Cancer Consortium (DKTK), Partner Site Frankfurt, Frankfurt, Germany; ^5^ Cardiopulmonary Institute (CPI), Frankfurt, Germany

**Keywords:** immunity, cancer, antigens, mouse models, tertiary lymphoid structures

## Abstract

The success of immunotherapy approaches, such as immune checkpoint blockade and cellular immunotherapy with genetically modified lymphocytes, has firmly embedded the immune system in the roadmap for combating cancer. Unfortunately, the majority of cancer patients do not yet benefit from these therapeutic approaches, even when the prognostic relevance of the immune response in their tumor entity has been demonstrated. Therefore, there is a justified need to explore new strategies for inducing anti-tumor immunity. The recent connection between the formation of ectopic lymphoid aggregates at tumor sites and patient prognosis, along with an effective anti-tumor response, suggests that manipulating the occurrence of these tertiary lymphoid structures (TLS) may play a critical role in activating the immune system against a growing tumor. However, mechanisms governing TLS formation and a clear understanding of their substantial heterogeneity are still lacking. Here, we briefly summarize the current state of knowledge regarding the mechanisms driving TLS development, outline the impact of TLS heterogeneity on clinical outcomes in cancer patients, and discuss appropriate systems for modeling TLS heterogeneity that may help identify new strategies for inducing protective TLS formation in cancer patients.

## Introduction

Cancer development is an evolutionary, multi-step process that can take several decades in humans. Throughout this period, the transformed cells continually interact with their local microenvironment, including the immune system. It is now firmly established that this interaction comprises several hallmarks of cancer that initially appear contradictory, as tumor-associated immune responses can either result in the rejection or progression of tumors (1, 2). On one hand, chronic inflammation triggered by environmental and lifestyle factors can give tissues enough plasticity to suppress their default tumor-suppressive nature and induce somatic mutations in local cells (3–5). Moreover, continuous low-grade inflammation may sustain tumor growth. Through this, several processes, including hypoxia, metabolic adaptations, interaction with dying cells or cellular debris, and negative feedback signals that physiologically limit autoimmunity during infection, educate immune cells to actively support tumor growth (4, 6, 7). On the other hand, altered self-cues, including neo-epitopes and stress-related cell surface molecules, can be recognized by the immune system, leading to tumor rejection (8–10). This process is likely the rule rather than the exception in humans, leading to the eradication of early cancerous lesions or keeping them in check. Tumors that survive these interactions often develop a highly immunosuppressive phenotype, enabling them to progress towards clinically relevant stages (8, 9, 11–15).

Evidence of active anti-tumor immunity was long debated but is now unchallenged due to clinical efficacy of immune checkpoint blockade (ICB), at least in some tumor entities (16, 17). Even in tumors where ICB shows low efficacy, bioinformatic analyses have demonstrated the prognostic and predictive relevance of the immune response in cancer patients (18, 19). Here, immune cell populations and activation states that correlate with positive or poor prognosis across different tumor types have been defined (20). Both, the density and anti-tumor activity of cytotoxic lymphocytes such as γδ T cells, CD8+ T cells, T helper 1 (TH1)-polarized CD4+ T cells, memory T cells or NK cells, as well as tumor-associated B cells, and some activated myeloid cell subsets, are associated with a favorable outcome for patients. In contrast, immunosuppressive myeloid cells including macrophages and immature myeloid-derived suppressor cells, as well as lymphocytes such as regulatory T cells (Treg) or TH17-polarized CD4^+^ T cells, often indicate poor prognosis (21–23). Given this association of immune quality with patient prognosis, mechanisms that shape protective versus tumor-promoting immunity are being intensively investigated. Besides counteracting tumor-promoting immunosuppressive cells, it is crucial to understand the characteristics determining if protective immunity is induced and persists in cancer patients. It is undisputed that cancer is a systemic disease and that the education of the immune system by cancer antigens in the periphery is an important requirement to induce anti-tumor adaptive immune responses both at baseline and during immunotherapy (24, 25). The generation of an efficient adaptive immune response against cancer typically occurs in secondary lymphoid organs (SLO), where antigens are presented to CD4+ T and CD8+ T cells by mature dendritic cells (DCs) (26, 27). However, when applying spatial analysis criteria to the determine prognostic role of immune cells in cancer, the concept emerged that adaptive immunity can, to a significant degree, also develop locally in newly formed TLS (27, 28). Understanding the principles guiding the formation of these structures and understanding their heterogeneity across cancer types may, thus, be instrumental to harness the full power of the immune system in the fight against cancer. This will be particularly important in patients that currently do not benefit from cancer immunotherapy.

## What are TLS

**Figure 1:**
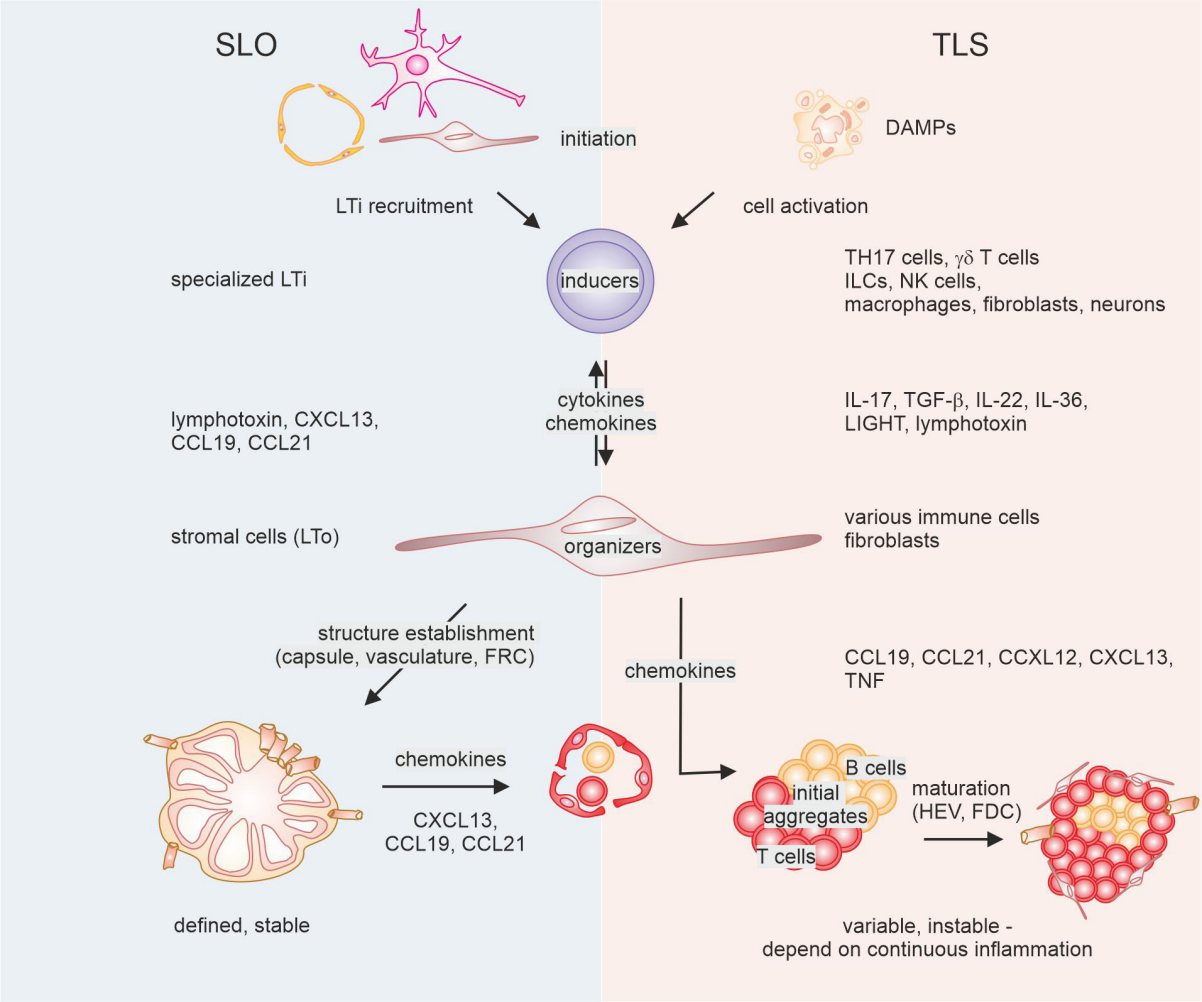
Mechanisms of secondary lymphoid organ (SLO) versus tertiary lymphoid structure (TLS) formation. The development of SLO (left) requires interaction between initial lymphoid tissue organizer (LTo) cells and lymphoid tissue inducer (Lti) cells, followed by activation of further LTO cells, and feed-forward recruitment of LTi cells. This ultimately leads to the establishment of a reticular and vascular structure that is populated by lymphocytes. In the case of TLS, diverse cells can fulfill the task of LTi cells, triggered by factors in the tumor microenvironment. These cells interact with diverse other cells that show LTO functionality and recruit lymphocytes. Eventually, such lymphoid aggregates may or may not be supplemented with follicular dendritic cells (FDC) and high endothelial venules (HEV) that are instrumental for the formation of a germinal center reaction. DAMPs, danger-associated molecular patterns; TH17, T helper 17; ILCs, innate lymphocytes; NK cells, natural killer cells; FRC, fibroblastic reticular cells, TGF, transforming growth factor; TNF, tumor necrosis factor.

TLS are ectopic hematopoietic aggregates that emerge in sites normally lacking lymphoid organs. TLS have certain developmental and structural similarities with SLO such as lymph nodes, the spleen, tonsils, Peyer’s patches, and mucosa-associated lymphoid tissues, but they also exhibit important differences (**Figure 1**). SLO are encapsulated, and therefore physically separated from their neighborhood, while TLS lack a solid capsule and are directly exposed to the inflammatory milieu in which they develop. Additionally, TLS development pathways seem to be more versatile. Unlike SLO, TLS form in response to chronic inflammation though a process called lymphoid neogenesis (29). This occurs in various disease settings including infection, anti-transplant immunity, autoimmunity and cancer, usually in an antigen-dependent manner. Importantly, TLS seem to necessitate sustained inflammation and may disassemble once inflammation resolves (30–32). Antigen-dependent immune responses within TLS, under the conditions described above, can be both protective and detrimental for the host, depending on the quality of the immune response within TLS. However, the latter seems to dominate during auto-immunity and anti-transplant immunity (33). Particularly in cancer, this contrast seems to hinge on the balance between regulatory T cells and effector lymphocytes, although this relationship is not yet fully understood (34–37). TLS exhibit varying cellular compositions, even within a single tissue, reflecting their maturation status, which appears to be disease-relevant, as outlined in more detail below (32, 38). TLS predominantly consist of B cells, T cells, DCs, follicular dendritic cells (FDCs), and sometimes high endothelial venules (HEVs). Their composition can range from loose clusters of lymphocytes and occasional myeloid cells to highly organized structures with distinct T and B cell zones and the formation of germinal centers (GCs), where high-affinity antibodies are generated (39). There is still much left to be understood regarding the processes guiding TLS formation and composition, and how the outcome of these processes is associated with disease activity and therapy response. Various challenges, such as limited human tissue availability, especially during the early stages of TLS formation, and the scarcity of robust and reproducible mouse models of TLS development, complicate investigations into these matters. These issues impede the design of longitudinal studies to precisely monitor the stages of TLS formation in cancer. Furthermore, there is still a lack of standardized markers useful for determining disease-relevant determinants of TLS heterogeneity. Additionally, while their role as disease biomarkers and their prognostic value for therapy response is evident, it is not fully understood if they directly impact on disease activity, particularly in cancer. Despite these limitations, TLS appear to exemplify the connection between auto-inflammation and anti-tumor immunity. Therefore, understanding TLS formation in cancer may not only benefit cancer patients by serving as biomarkers and therapeutic targets but may also be essential in modulating their formation during infection and chronic inflammatory reactions. In the following pages, we will summarize the current knowledge concerning TLS development, the impact of TLS heterogeneity on cancer development and therapy, and outline and discuss suitable models to study lymphoid neogenesis in cancer.

## TLS development

To understand the degree of heterogeneity observed in recent studies regarding TLS formation, a comparison with SLO formation can be instrumental. While there are evident similarities in the sequential events leading to SLO and TLS formation, the diversity at each stage is notably amplified in the case of TLS 31, 32) (**Figure 1**). This is illustrated by findings showing that TLS can develop in mice and humans, even in the absence of SLO (40–42).

Even though SLO formation is not uniform due to variations in the tissue environment where they develop, common patterns haven been identified by studying genetically modified mice lacking SLO (43–46). Elaborate mechanistic hypotheses explaining SLO development have been extensively reviewed elsewhere (47–49). In brief, SLO formation requires a stepwise interaction between lymphoid tissue organizer (LTo) and lymphoid tissue inducer (LTi) cells. The latter belong to the innate lymphocyte lineage and differentiate from CD3- CD4+/- CD127+ CD45+ innate lymphoid progenitors in the fetal liver, regulated by the nuclear hormone receptor retinoic acid related orphan receptor γt (RORγt) and the transcription inhibitor Id2 (45, 50). During embryogenesis, LTi cells are initially recruited to lymph node Anlagen by CCL21-expressing lymphatic endothelial cells and/or mesenchymal cells that produce CXCL13 under the influence of retinoic acid, which can itself be produced by nerve cells (46, 51). Juxtacrine signaling between lymphotoxin expressed on LTi cells and the LT-β receptor expressed on LTo cells further induces chemokine production and adhesion molecule expression by LTo cells, leading to the recruitment of more LTi cells. This initiates a positive feedback loop, resulting in the remodeling of the lymphatic vasculature (52) and the stromal compartment, along with the formation of a capsule. Only after these structures are formed, lymphocytes are abundantly recruited through newly formed high endothelial venules (HEV), and a stable cellular architecture is established (31, 51).

The initial stages of TLS formation parallel SLO development in the sense that interaction between inducer and organizing cells, which then recruit lymphocytes, appears critical. However, both inducer as well as organizing cells are not as strictly defined as during SLO generation. In TLS, the role of organizing cells is often assumed by activated local fibroblasts producing chemokines such as CCL19, CCL21, CXCL12, and CXCL13, along with adhesion molecules that recruit B cells and T cells to form initial loose aggregates (31, 32, 53, 54). Moreover, other cells, including macrophages, DCs, and CD8+ T cells, have been shown to produce CXCL13 for recruitment of lymphocytes as well (55–57). Additionally, CCL19+ DCs have been correlated with the presence of TLS and other lymphoid aggregates in breast cancer (58). The activation of these diverse LTo-like cells in TLS can occur *via* various sources and mediators other than specialized innate lymphocyte LTi cells. Several mediators derived from such cells have been identified, although what triggers the activation of LTi-like cells initially remains largely unknown. Factors in the tumor micromilieu, such as DAMPs and mediators from dying cells, likely play a role. Various cytokines such as IL-13, IL-17, IL-22, and type 1 interferons from cells substituting for LTi can activate stromal cells to support TLS formation (53, 59–62). In colorectal tumor models, IL-36 production by macrophages and endothelial cells has been shown to be involved in TLS formation (63, 64). IL-36 activates fibroblasts during intestinal inflammation (65). Whether IL-36 acts *via* fibroblasts to promote TLS development is yet to be determined. Conversely, fibroblasts were observed to induce CXCL13 in T cells through TGF-β production (66, 67).

Finally, similar to SLO formation, activation of the LT-β receptor on stromal cells by both, lymphotoxin and and alternative ligand, LIGHT, promote TLS development (61, 68–73). However, the early events during lymphoid neogenesis can occur independently of LT-β receptor signaling (53, 61, 74). Similar to SLO, signals from nerve cells may play a role in activating stromal cells (74). LT-β receptor signaling seems particularly necessary for later stages of TLS maturation. For instance, a combination of antiangiogenic and immune-modulating therapies provoked the generation of HEV *via* lymphotoxin/LT-β receptor interaction (75). The formation of HEV is viewed as a sign of TLS maturation (31). However, the maintenance of HEV can occur independently of LT-β receptor, requiring the presence of T and NK cells and/or cytokines such as IL-36 (63, 75, 76). Also the generation of FDCs, involved in GC reactions for optimized antibody production (77), was found to depend on LT-β receptor signaling (78).

These findings indicate that key principles and cellular interactions are similar between TLS and SLO formation. The relative heterogeneity of involved cells and mechanisms for TLS formation in cancer may still be underestimated, given the diversity of immune environments even within a single tumor. Although TLS were shown to form 3D intercommunicating networks in colorectal tumors, individual networks within a single tumor exhibited different cellular compositions (38). Given the multitude of signals that are able to induce TLS formation, the question remains why TLS are not always formed during carcinogenesis. One explanation would be the presence of TLS-restricting signals in cancer, as is the case under homeostatic conditions. Identifying such signals in the future may open new avenues for TLS induction. The potential predictive and therapeutic value of such strategies is summarized in the following chapters.

## TLS in cancer

The majority of current literature suggests that a high density of TLS is associated with favorable outcomes in solid tumors. However, some investigations have identified TLS density as a marker of disease progression with adverse prognostic implications. There is a lack of systemic studies to define the heterogeneity of TLS, further exacerbated by the absence of uniform scoring criteria (79), hampering the evaluation of TLS in cancer. To perform a rigorous assessment of TLS, certain elements should be carefully considered at the very least: the composition and maturation of TLS, the size of TLS, the density of TLS, and the location of TLS.

## The cellular heterogeneity of TLS

Typically, TLS are believed to promote anti-tumor immunity by recruiting immune cells and activating adaptive immunity. As a result, TLS are highly correlated with improved survival outcomes in many cancers, such as breast cancer (80–82), hepatocellular cancer (HCC) (83), colorectal cancer (CRC) (84, 85), melanoma (86), gastric cancer (87, 88), head and neck squamous cell cancer (HNSCC) (89, 90), lung cancer (79, 91) and sarcoma (92). However, it has also been reported that TLS show little correlation with overall survival or are even correlated with high pathologic grade and poor outcomes in malignant diseases, such as breast cancer (80) and HCC (93), posing an obvious contradiction to the previously mentioned studies in these entities. Recent studies indicated that the discrepancy was attributed to the heterogeneity and spatial distribution of TLS in these tumors. As mentioned earlier, unlike SLO, in most tissues, TLS are characterized by CD20+ B cells (B-cell zone) surrounded by CD3+ T cells (T-cell zone), with no capsular involvement (94, 95). This specific anatomical structure facilitates direct interactions between immune cells and the tumor microenvironment. The composition of immune cells in TLS may vary in different tumors or even within single tumors (38). The complex lymphoid aggregates which make up TLS are composed of various immune cells and stromal cells. The immune cells include B cells, T cells, FDCs, and myeloid cells such as other DC subsets and macrophages. The stromal cells, such as follicular reticular cells, fibroblasts and vascular cells (e.g. forming HEVs), are believed to maintain the integrity of the non-capsulated structure and mediate the recruitment of immune cells. We will primarily focus our discussion on the role of B cells, T cells and HEVs from TLS in solid tumors.

## B cells and TLS maturation

Extensive clinical and experimental evidence suggests that B cells play a crucial role in the cancer microenvironment, indicating a positive correlation with patient outcomes in various tumors (96–99). It is speculated that B cells in TLS also play a beneficial role by mediating antigen presentation, facilitating T cell activation and development, and producing tumor-specific antibodies in GC reactions, while contributing to GC formation themselves. GC formation appears as a potent criterion for predicting if TLS are prognostically relevant, serving as a marker for TLS maturation. The maturation of TLS is believed to be essential for activating immunity in cancer and indicating immune therapy efficiency in solid tumors (100). The maturation of TLS has been categorized into three phases: early TLS (eTLS, lymphocyte aggregates), primary follicle-like TLS (pTLS, immature TLS without GCs), secondary follicle-like TLS (sTLS, well-developed lymphoid structures with GCs) (101, 102). An immunostaining panel, including CD20, CD21 and CD23, has been devised to identify the status of TLS in metastatic melanoma. Mature TLS were defined as the presence of CD20+, CD21+ and CD23+ lymphoid aggregates (101). Recent mass cytometry studies confirmed this classification: early lymphoid aggregates lacking organization or GC function were CD20+CD21-CD23-, non-GC TLS were CD20+CD21+CD23- (organized but lacked GC functionality), and GC-containing TLS showed GC organization and functionality associated with the expression of all three markers (CD20+CD21+CD23+) (103). CD23 was even suggested as a useful single marker for mature TLS, at least in breast cancer (104). Interestingly, in a lung cancer cohort, lymph node (LN) metastasis was associated with reduced B cell infiltration and fewer GC formations in TLS. GC+ TLS, rather than non-GC TLS, predicted better outcomes in lung cancer (105). So far, the maturation status of TLS, particularly GC formation (38), has been investigated in various solid tumors, such as esophageal cancer (102), CRC (106), lung cancer (107) and melanoma (101, 108), with the presence of GC+ TLS predominantly associated with a favorable outcome in cancer patients. The relevance of GC formation indicates a strong contribution of B cells to the beneficial impact of TLS in cancer. Although growing evidence suggests an important role of B cells in anti-tumor immunity and immunotherapy (109–111), the role of B cells in TLS towards clinical relevance is still understudied. Helmink and colleagues found that in an immune checkpoint blockade (ICB) trial in melanoma patients, B cells and TLS were more abundant in responders than non-responders. Similar B cell enrichment together with TLS abundancy pattern were validated in a renal cell carcinoma (RCC) ICB trial (109). In a lung adenocarcinoma cohort, a TLS-linked B-cell signature predicted beneficial outcomes in patients treated with PD-1 or PD-L1 inhibitors (112). In summary, mature TLS correlated with B cell presence appears to be involved in anti-tumor immunity and may confer beneficial immunotherapy response and favorable prognosis, although causality remains to be determined. Further studies investigating B cell heterogeneity in TLS may yield even better markers compared to the three-gene (CD20, CD21, CD23) signature. Hereby, establishment of a memory B cell response is likely required to confer long-lasting protection (105).

## Divergent role of T cells in TLS

In addition to B cells, the presence of TLS is highly associated with tumor-infiltrating T cells (113). These cells have been extensively studied in the context of basic tumor biology and treatment response, especially in cancers such as CRC (114, 115), breast cancer (116, 117), and lung cancer (118–120). It is well documented that intraepithelial CD8+ T cells, in particular, are associated with a favorable prognosis in solid tumors, including ovarian cancer (121), breast cancer (122), and CRC (123). Additionally, tumor-infiltrating T cells in the stroma also correlate with improved survival in cancer patients. A standardized methodology for assessing stromal tumor-infiltrating T cells in breast cancer was first proposed in 2014 by the International TILs Working Group (124). The model was subsequently modified to evaluate tumor-infiltrating T cells in other cancers as well (125, 126). These studies demonstrated that the presence of tumor-infiltrating T cells remained a powerful predictive factor for most malignancies. Chaurio and colleagues identified that TLS formation was dependent on the CXCL13 pathway in CD4+ T cells, with blocking CXCL13 hindering TLS assembly and subsequently promoting tumor growth (127). Additionally, CD8+ T cells were found to be an important source of CXCL13, mediating immune cell recruitment into TLS and enhancing the sensitivity to immunotherapy in lung cancer (56). Similarly, in another six cohorts of human cancer, a high density of CD8+ tumor-infiltrating T cells was associated with increased B cell recruitment and TLS formation (128). These studies emphasized that T cells play a crucial role for TLS formation and anti-tumor immunity, two phenomena which may, but do not necessarily have to be functionally connected. However, in a cohort of advanced CRC (129), a high ratio of tumor-infiltrating T cells in TLS was associated with tumor recurrence, suggesting a potential deleterious role of tumor-infiltrating T cells in tumor progression. Interestingly, in advanced lung adenocarcinoma, Tregs in TLS were found to suppress anti-tumor immune responses, despite TLS promoting T cells trafficking and activation of the tumor microenvironment (34). In non-small cell lung cancer patients, stromal Tregs suppressed the proliferation of other CD4+ T cells, and a high density of stromal Tregs and Treg cells in TLS correlated with poor outcomes (36). In a prospective study on sarcoma, high Treg numbers in TLS predicted poor responses to ICB treatment, and patients with Treg-enriched TLS had worse survival outcomes (130). These results suggest that not only the functional polarization of tumor-infiltrating T cells *per se* but also within TLS is an important criterion in tumor immunogenicity during tumor progression. Comprehensive quantification of tumor-infiltrating T cell subsets in TLS should be considered to evaluate their prognostic value in different cancer types. Additionally, the phenotypes and functional properties of suppressive Tregs in TLS and their potential association with TLS maturation require further investigation. Interestingly, in tumors of pancreatic ductal adenocarcinoma (PDAC) patients that had received neoadjuvant chemotherapy, a lower proportion of B cells and a higher proportion of regulatory T cells within intratumoral TLS were observed. These TLS were smaller with a reduced maturation level and immune cell activation, leading to a lack of prognostic value of TLS presence in this cohort. (131). Importantly, not only Tregs but also T cell exhaustion phenotypes may be linked to TLS maturation. In breast cancer, while tumors with enhanced exhausted-like T cells contained higher levels of CXCL13-expressing T cells, their presence correlated with more immature rather than mature TLS (132).

## Role of HEV in TLS formation

HEVs play an active role in the formation of TLS, boosting anti-tumor immunity by facilitating immune cell trafficking from the peripheral blood to the tumor microenvironment (133–136). A recent study demonstrated that around 40% of lymphocytes entered tumor sites through HEVs during ICB treatment, highlighting HEVs as the primary route for lymphocyte entry into tumor lesions (137). Furthermore, HEVs have been identified as a positive factor for immunotherapy and have shown correlation with improved survival outcomes for melanoma patients. Another study in melanoma and NSCLC indicated that a high HEV score was among patients responding better to ICB, supporting the significance of HEV as an important prognostic factor for immunotherapy (75). However, HEVs also promote tumor metastasis by providing exit points for disseminating tumor cells in murine models and human cancers (138–141). LN metastasis is among the strongest prognostic indicators for clinical outcome of malignant tumors. Regional LN irradiation improves the survival outcome for both early-stage and advanced tumors (142, 143). A recent study indicated that HEV-associated genes were not only linked to high aggregates of T cells and B cells in TLS but also correlated with longer survival in breast cancer (144). Zhan and colleagues performed immunohistochemistry on 203 CRC samples, categorizing them into high and low HEV/TLS groups based on the average area of HEV/TLS (145). A high proportion of HEVs in TLS was associated with a favorable prognosis of CRC suggesting enhanced anti-tumor immunity in the high HEV/TLS groups. HEVs remain an important and complex component in TLS, and further research is necessary to understand the mechanisms of immune cell trafficking and tumor cell dissemination through HEVs. Moreover, studies addressing the molecular mechanisms of HEV generation in TLS are required. In conclusion, markers for the cellular composition of TLS that are linked to TLS maturation and offer insights into their prognostic and therapeutic potential are emerging. However, to utilize to full potential of these markers, further issues need addressing, including standardized protocols for TLS quantification.

## Quantification of TLS

Numerous studies have attempted to investigate the size and number of TLS that predict outcomes in solid tumors. However, most studies face limitations due to inconsistent definitions of TLS, distinct quantification methods, retrospective approaches, and single-center experiences. Consequently, the development of an integrative methodology and standardized scoring system to identify the size and density of TLS remains a subject of debate. Pathological evaluations, including Hematoxylin and Eosin (H&E) staining (102, 146–151), fluorescence immunohistochemistry (f-IHC) (53, 152) and multiplex IHC (101, 109), have been shown to be the most straightforward and reliable methods to quantify TLS in tumors. Of note, the majority of studies involved both quantitative and qualitative analyses (153–156). Rakaee and colleagues established three models to quantify TLS in NSCLC. The semi-quantitative method categorized the TLS into four groups based on the number of TLS in the tumor. The quantitative method counted the absolute number of TLS in the tumor and adjacent tissues. The final model compared the GC+ TLS group with the GC- TLS group (79). In a human melanoma study, the counts and area of TLS were normalized to tissue area for quantifying the density of TLS in tumor sections (157). In a cohort of 1924 gastrointestinal cancer patients, a machine-learning model was developed based on histopathology images. The overall TLS score was defined as the sum of a weighted linear eTLS area, pTLS area and sTLS area normalized by tumor area (155). Considering the relative value of maturation of TLS, the final weights of TLS were optimized by Cox regression analysis in the TCGA stomach adenocarcinoma cohort. Patients with high TLS scores exhibited significantly improved overall and disease-free survival compared to those with low TLS scores.

Recently, large scale gene expression analyses, such as RNA-sequencing and spatial transcriptomics, have been implemented to study the landscape of TLS in cancer. TLS-signature genes, including CD79B, CD1D, CCR6, LAT, SKAP1, CETP, EIF1AY, RBP5, and PTGDS, were identified through significance analysis of microarrays, underlining the importance of TLS in melanoma metastasis and immunotherapy (158). High expression of these nine-gene TLS signatures correlated with better overall survival and positive responses to ICB in melanoma. More importantly, the nine-gene TLS gene signature has been recently validated in high-grade serous ovarian cancer, demonstrating better disease-free survival for patients with high TLS scores (159). In CRC and metastatic CRC, 12 chemokines including CCL2, CCL3, CCL4, CCL5, CCL8, CCL18, CCL19, CCL21, CXCL9, CXCL10, CXCL11, and CXCL13, were closely associated with TLS formation. The geometric mean of the above 12 genes was calculated to evaluate TLS in tumor (156, 160). Single cell RNA-sequencing combined with bulk RNA-sequencing of HNSCC revealed CXCR3, CCR7, CCR6, CXCR5, and CCR1 as TLS-associated chemokine receptors, largely dentifying the receptor counterparts to the identified chemokine signature (161). Similar TLS gene signatures have been defined for other solid tumors to predict survival and responses to immunotherapy (89, 162–164). Presently, there is no standardized methodology for quantifying TLS in cancer. However, it is believed that a combination of histology and gene expression analysis would provide a better understanding of TLS composition and function in cancer. Emerging markers, such as the presence of Tregs or HEVs, might also be considered for such analyses. Yet, distinguishing “high” and “low” or defining a specific “cut-off” point in the data is often not objective and challenging to apply uniformly across different sites.

## Location of TLS

Besides precise quantification, the spatial distribution of TLS within tumors might add another layer of complexity. Hereby, TLS can be distributed across tumor nests (T-TLS), the peritumoral area (P-TLS) and tumor stroma (S-TLS) (95). The prognostic value of TLS density in solid tumors has been shown, though inconsistently. Discordance in prognosis may be partially due to different spatial distributions of TLS. However, results across multiple studies were mixed: some studies demonstrated that P-TLS rather than T-TLS were positively correlated with favorable prognosis, while others showed contradictory results. Moreover, the exact delimitation of the three regions remains controversial. An evaluation of the prognostic value of TLS in patients with non-metastatic CRC revealed that high P-TLS contributed to favorable outcomes for patients with CRC, while T-TLS did not significantly correlate with clinical outcomes. The TLS and tumor stroma percentage, representing S-TLS, showed a negative correlation with overall survival for patients (85). Conversely, in CRC liver metastasis, P-TLS were negatively correlated with relapse-free and overall survival, whereas T-TLS were significantly correlated with better outcomes (156). Similarly, Ding et al. found in a cohort of 962 intrahepatic cholangiocarcinomas (CCA) patients from three cancer centers across China that T-TLS were associated with a favorable prognosis, whereas P-TLS indicated a worse outcome (165). Additionally, a high T-TLS score correlated with better prognosis and response to immunotherapy in CCA patients (166).For breast cancer patients, the presence of P-TLS was linked to worse clinical outcomes (167). It has been reported that T-TLS indicated a lower risk of early recurrence in HCC (83), and an enhanced response of ICB in resistant tumors (168). In summary, the majority of literature supports the notion that T-TLS are associated with positive prognostic effect in cancer. Notably, variable definitions and cut-offs may cause confusion when discussing P-TLS and S-TLS. Studies have tended to conflate P-TLS and S-TLS, resulting in limited reports on S-TLS in tumor-immune contexture. However, definitions of P-TLS and S-TLS in cancers remain underexplored. Researchers have adopted a similar definition of P-TLS in CRC, defining the area up to 7 mm from the infiltrative edge (106, 156, 169). Sofopoulos and colleagues defined P-TLS in the area 5 mm away from the tumor invasive margin (167). In the CCA cohort, the peri-tumor region was defined as a normal tissue area 5 mm away from the tumor edge (165). In HCC, the peritumoral area was also considered as the region 5 mm distant from the invasive tumor border (170). These studies again suggest that P-TLS do not always play a protective role in solid tumors, which can be attributed to factors such as tumor types, heterogeneity, status, and staging.

In summary, T-TLS provide an important niche for supporting anti-tumor immunity and are associated with improved clinical outcomes in many tumors. However, the value of P-TLS and S-TLS in determining prognosis remains a subject of debate. Standardized scoring systems of T-TLS, P-TLS and S-TLS are critical to evaluate their functions across different cancer types and cancer stages. Moreover, the reasons for the association of spatial distribution of TLS with clinical outcome in cancer patients need to be studied. The influence of the highly suppressive stromal microenvironment may be at work. Recent multiplexed 3D reconstructed imaging in CRC has revealed that TLS can form interconnected, graded networks, suggesting communication in such larger networks. Additionally, within a single tumor, these networks show diverse cellular compositions (38). Thus, not only the 2D localization but also the interconnectedness of TLS might become an important criterion in the future. Whole-body imaging techniques applied to analysis of TLS in mouse models might aid in studying this aspect (171). Finally, TLS have been shown not only to form at primary but also secondary metastatic sites, which likely significantly affects patient prognosis.

## TLS in metastatic cancers

Metastases continue to be the primary cause of cancer mortality, accounting for nearly 90% of cancer-related deaths (172). Studies have shown the formation of TLS at metastatic sites such as the liver and lungs (173). While the majority of literature supports the theory that TLS in metastatic sites contribute to anti-tumor immunity (27), it remains unclear whether there is TLS heterogeneity between primary tumors and metastatic sites. Reliable data concerning the role of TLS at metastatic sites are scarce. In a cohort of CRC and RCC metastases, metastasis-associated TLS exhibited a high degree of similarity with TLS in primary tumors, including their density and cellular composition (174). This suggests either a dominant role of the primary tumor cells in TLS formation, or alternatively suggests a prominent role of systemic tumor-associated immunity in TLS development at different sites. The former assumption is supported by the fact that CRC lung metastases exhibited more abundant TLS in lung stroma compared to RCC lung metastases, which was in line with increased TLS formation in primary CRC lesions. Furthermore, both CRC and CRC lung metastases displayed a significant increase of CD3+, CD8+ T cells, and DCs in TLS. In CRC liver metastases, TLS at the tumor-liver interface, characterized by CD45+CD20+ B cell aggregates, indicated a reduced risk of tumor relapse and a favorable overall survival (175). Similarly, Ahmed and colleagues found that TLS at invasive margins, rather than tumor lesions, were correlated with better survival in CRC liver metastases (176). In a cohort of patients with omental metastases from high-grade serous ovarian cancer, B cells in lymphoid aggregates showed enhanced anti-tumor immunity, particularly boosted by chemotherapy (177). In cutaneous melanoma metastases, patients with positive TLS exhibited improved overall survival. Interestingly, the maturation of TLS was not related to survival outcome, while CD20+CD21+ B cells in TLS correlated with a worse prognosis in metastatic melanoma (101). In a cohort of patients with breast cancer metastases consisting of 355 metastatic samples from the lung, liver, brain, and ovary, no TLS were found in brain and ovarian metastases. The presence of TLS at metastatic sites was an independent factor for a favorable prognosis (178). However, two studies on lung metastases from CRC indicated that the presence of TLS at metastatic sites had no prognostic value (84, 179). These studies suggest that the complex immune contexture of TLS in metastatic cancers is determined by both primary tumor and metastatic lesions. While the majority of data indicate that TLS play a beneficial role in metastatic cancers, some uncertainties and controversies persist. Taken together, TLS formation appears to be relevant in the tumor microenvironment of metastatic cancers. Further research is required to enhance our understanding of the mechanisms behind TLS formation and their action in metastatic cancers, and the interrelationship between TLS at primary and secondary sites.

## Clinical trials related to TLS

A range of clinical trials have underscored the viability of immunotherapy in enhancing patient outcomes, encompassing ICB, cancer vaccines, adoptive cellular therapy, and small molecule-based immunomodulators (180, 181). It is well documented and validated that the combination of immunotherapy and chemotherapy can lead to an improved pathological complete response and enhanced surgical feasibility post neoadjuvant treatment (182–185). Furthermore, adjuvant chemotherapy and immunotherapy substantially improved postoperative DFS and have been considered as a standard of care for select patients (186–188). The induction of TLS during chemoimmunotherapy and its positive effect on patients has raised particular interest (189, 190). However, the significance of TLS in cancer treatment has long been overlooked because studies typically focused on a single cell population, such as lymphocytes, macrophages, and fibroblasts, rather than lymphoid aggregates. Although much remains unknown about TLS in cancer treatment, recent clinical trials suggest TLS as a crucial participant in the tumor microenvironment and demonstrate a close correlation between the presence of TLS and sustained clinical benefits (**Table 1**) (198, 201, 202).

**Table 1:** Clinical trials related to TLS.

Study duration	Patient No.	Study type	Phase	Trial ID	Treatment	Cancer type	Reference
Concluded trials							
2008.07-2019.02	87	Interventional	phase 2	NCT00727441	GVAX vaccine	PDAC	(191)
2008.11-2023.08	75	Interventional	phase 1	NCT00788164	HPV vaccine	cervical intraepithelial neoplasia	(192)
2014.09-	45	Interventional	phase 2	NCT02259621	neoadjuvant anti-PD-1	NSCLC	(193)
2018.01-2021.09	54	Interventional	phase 1	NCT03387761	neoadjuvant anti-PD-1 and anti-CTLA-4	urothelial cancer	(190, 194)
2013.07-2019.04	18	Interventional	phase 1	NCT01804712	neoadjuvant anti-CD20	prostate cancer	(195)
2016.03-2022.06	29	Interventional	phase 1	NCT02626130	anti-CTLA-4 and cryoablation	Metastatic RCC	(196)
2018.05-2021.10	15	Interventional	phase 1	NCT03299946	neoadjuvant anti-PD-1 and tyrosine kinase inhibitor	HCC	(197)
2015.06-2021.08	227	Interventional	phase 2	NCT02406781	anti-PD-1 and chemotherapy	sarcoma	(92)
2016.11-2022.03	45	Interventional	phase 2	NCT02901899	anti-PD-1 and chemotherapy	ovarian cancer	(198)
2016.01-2021.06	730	Interventional	phase 2	NCT03013335	anti-PD-1	Metastatic RCC	(199)
2017.06-	101	Interventional	phase 2	NCT03158129	neoadjuvant anti-PD-1, anti-CTLA-4 and chemotherapy	NSCLC	(200)
2016.05-2022.09	24	Interventional	phase 2	NCT02592551	neoadjuvant anti-PD-L1 and anti-CTLA-4	malignant pleural mesothelioma	(201)
2015.11-2019.06	87	Interventional	phase 1/2	NCT02541604	anti-PD-L1	multiple tumors from pediatric patients	(202)
Ongoing trials							
2023.09-	102	Interventional	phase 2	NCT05888857	anti-PD-1 and anti-CTLA-4	solid tumors	N.A.
2024.01-	120	Interventional	phase 2	NCT06084689	MDM2 inhibitor and anti-PD1	solid tumors	N.A.
2022.03-	80	Interventional	phase 2	NCT04874311	anti-PD-L1 and chemotherapy	sarcoma	N.A.
2022.12-	66	Interventional	phase 2	NCT04968106	anti-PD-1 and chemotherapy	sarcoma	N.A.
2020.02-	67	Interventional	phase 2	NCT04095208	anti-PD-1 and anti-LAG-3	sarcoma	N.A.
2021.07-	173	Interventional	phase 2	NCT04705818	anti-PD-L1 and EZH2 inhibitor	solid tumors	N.A.

MDM2, mouse double minute 2 homolog; LAG-3, lymphocyte-activation gene 3; EZH2, enhancer of zeste homolog 2; N.A., not applicable.

Lutz and colleagues conducted a phase 2 study of neoadjuvant and adjuvant vaccines with irradiated, granulocyte-macrophage colony-stimulating factor–secreting, allogeneic PDAC vaccine (GVAX) +/- low dose cyclophosphamide. They found that TLS formed in 85% of participants two weeks after vaccination. Inhibition of the Treg pathway and activation of the IL-17 pathway within the TLS were associated with improved survival for PDAC patiens (191). Notably, PDAC with intratumoral TLS formation exhibited an enhanced PD-1/PD-L1 pathway, suggesting that vaccine-treated PDAC was converted into an immunogenic tumor, potentially benefitting from anti-PD-1/PD-L1 ICB. Similarly, in a phase 1, open-labeled clinical trial on high-grade cervical intraepithelial neoplasias, patients received a DNA vaccine targeting HPV16 E7, followed by a boost injection of vaccinia targeting HPV16 and HPV18 E6 and E7. Abundance of organized TLS was noticed in the proximity of vaccinated intraepithelial lesions rather than unvaccinated lesions (192). More importantly, histological alterations were closely associated with a gene signature of immune activation, indicating the induction of a robust tissue-localized immune response. TLS formation was also observed in neoadjuvant chemoimmunotherapy in patients with operable malignancies. A pilot study of metastatic RCC showed that tremelimumab with and without cryoablation increased TLS formation in patients with clear cell histology compared with baseline (196). In another study using nivolumab in patients with metastatic RCC, a significant enrichment of TLS was observed in responders rather than non-responders, showing a trend for improved outcomes (199). Similarly, Ho et al. reported the results of a single-arm phase 1b trial of neoadjuvant cabozantinib and nivolumab in patients with locally advanced HCC. They confirmed that enriched TLS formation was associated with improved responses to neoadjuvant treatments (197). Cascone and colleagues designed a neoadjuvant clinical trial comparing nivolumab + chemotherapy given as a dual therapy or in combination with ipilimumab as a means to estimate the major pathological response in NSCLC patients. Among 22 patients in the dual-therapy group, 7 patients exhibited a major pathologic response (32.1%), whereas 11 patients had a major pathologic response in the triple-therapy group consisting of 22 patients (50%). Increased TLS formation was observed in the triple-therapy group, suggesting immune activity and a close correlation with enhanced pathologic response (200). Since the major pathologic response was defined as more than 90% tumor regression in the context of chemotherapy, Cottrell et al. proposed to establish novel immune-related pathologic response criteria that highlighted the quantification of TLS in neoadjuvant chemoimmunotherapy (193). Importantly, the new criteria were shown to be reproducible and consistent among pathologists. In a single-arm trial of advanced urothelial cancer, 24 participants were treated with 2 doses of ipilimumab and 2 doses of nivolumab, and were evaluated for surgical resection within 12 weeks after initiation of neoadjuvant treatment. A pathological complete response occurred in 46% patients, and TLS were observed upon immunotherapy in responding patients (190). In-depth analysis of the immune contexture of resected samples was conducted to assess the significance of TLS for predicting responses to immunotherapy in urothelial cancer. Compared with deeper TLS, superficial submucosal tissue was characterized by enhanced T-helper cell infiltrations, abundant early TLS, and rare occurence of mature TLS. Interestingly, an increased enrichment of Foxp3+ T-cell-low TLS cluster was observed in unresponsive tumors, whereas a high abundance of macrophage-low TLS cluster was identified in treated tumors (194). The heterogenic TLS clusters were considered as promising biomarkers for predicting responses to immunotherapy in urothelial cancer. Furthermore, the composition of TLS was altered after neoadjuvant immunotherapy in patients with high-risk prostate cancer. Both B and T-cell densities in TLS were significantly reduced in patients receiving one dose of rituximab before prostatectomy (195). The studies mentioned above determined TLS formation as one among various parameters. Notably, a multi-cohort phase 2 study of pembrolizumab combined with chemotherapy in patients with sarcoma specifically assessed the prognostic significance of TLS and showed substantially improved outcomes in a cohort of sarcoma patients positive for TLS. The 6-month non-progression rate and objective response rate were 40% and 30%, respectively, in the cohort of TLS-positive patients, which were approximately 10-fold higher than in all-comer cohorts (92). Undoubtedly, the presence of TLS may provide a new perspective to assess the response to chemoimmunotherapy and the prognosis of patients. However, determining TLS formation in cancer patients remains a long way from being adopted in clinical practice, despite the evidence that it is intrinsic to immune responses to neoadjuvant and adjuvant treatment in clinical trials. A limited number of clinical trials specifically investigating the role of TLS in cancer are currently ongoing or under development (**Table 1**). The data emerging from these studies are expected to facilitate the clinical translation of TLS towards patient management. Future trials should consider the complex aspects of TLS biology outlined above, including TLS composition, size, maturation, localization, interconnectedness and appearance at different sites. However, determining which of these above-mentioned parameters are the most promising will need to be established in pre-cinical studies, for which reliable mouse models for studying TLS formation are needed. The currently available models and their suitability are discussed in the following paragraphs.

## Modelling TLS formation

The arsenal of experimental models for studying cancer has significantly expanded in recent years, thanks to improved mathematical and bioinformatics modeling tools and human tissue cultures, such as tumor slice cultures or patient-derived organoids (203–205). However, these techniques currently have clear limitations when attempting to model the spatiotemporal and cellular complexity of TLS formation. For instance, modelling TLS in organoid cultures would not only require the population of these cultures with patient-derived PBMCs to avoid alloreactions, but also a pre-population with fibroblasts and potentially endothelial cells. While these steps have been realized individually, combining them poses major logistical and technical challenges (206). Therefore, we focus our attention on mouse models of TLS formation in the following chapters.

The generation of murine models that mimic the development of solid cancer is complex. If the tumor cells exhibit rapid growth kinetics, the tumor burden will likely reach unacceptable levels before TLS can develop. Conversely, if the mutational burden is low and tumors develop slower, the availability of tumor antigens necessary for activation of the adaptive immune system and the development of TLS is limited (207). Nevertheless, there are autochthonous tumor models with spontaneous development of carcinomas, including mature TLS within the tumor or in close proximity, resembling lung adenocarcinoma (208, 209), PDAC (210), and HCC (93). To mimic the human situation, such genetically engineered mouse models (GEMMs) contain multiple mutations, such as overexpression of oncogenes (e.g. Kras) or deletion of tumor suppressors (e.g. p53), which are also present in the corresponding human cancer. Most importantly, tumor growth in these models can be modified *via* TLS-associated factors. These models are also suitable for developing new immune-based therapy options utilizing the power of TLS, including sensitizing tumors to ICB or CAR-T cell therapy (73). Autochthonous animal models that allow a stringent analysis of organized TLS formation for CRC, breast cancer, and melanoma have not been reported so far.

Besides autochthonous models, orthotopic tumor models have been used to study TLS development. This involves the injection of cancer cells from murine or human origin into recipient mice, either WT or immunodeficient, specifically into the tissue the tumor originated from. A more frequent approach is the heterotopic transplantation of tumor cells into recipient mice, such as the s.c. or i.p. injection of B16 melanoma cells. However, evidence that the localization or transplantation site of a tumor matters emerged from the finding that orthotopic transplantation of murine lung adenocarcinoma cells into C57BL/6 mice resulted in the activation of the adaptive immune system, while s.c failed to induce activated CD8+ T cells (208). Moreover, s.c. transplantation of tumor cells led to the accumulation of immune cells but did not allow the formation of mature TLS (210). GEMM models have already enabled the identification of factors necessary for TLS formation including lymphotoxin and CXCL13 (209, 211). Additionally, genetic modification of tumor cells *in vitro* enables the adaption of (orthotopic) transplantable models to a specific question, for example, the addition of artificial antigens such as OVA to increase immunogenicity and/or TLS formation. Through these means, tailored mouse models specifically for investigating TLS biology may be developed in the future.

## TLS in lung adenocarcinoma models

The investigation of lung adenocarcinoma (LUAD) using GEMMs showed the necessity for multiple genetic alterations to resemble the human disease, including TLS formation. Initially, a mouse model with a Lox-Stop-Lox Kras G12D mutation was used in combination with an intranasal or intratracheal application of an adenovirus or lentivirus containing a Cre recombinase for targeted mutation in the lung (K mice) (212). DuPage et al. observed enhanced tumor growth upon an additional deletion of p53 (KP mice) within 2-3 weeks (213, 214). Using the KP mice, Joshi et al. detected low- and high-grade lung adenocarcinomas after 20-24 weeks, but without TLS formation or TLS precursor (34). After depletion of regulatory T cells, the tumor burden was significantly increased, coinciding with TLS detection, emphasizing the significance of intrinsic anti-inflammatory mechanisms. In 2022, Boumelha et al. further developed the KP mice by introducing an overexpression of the APOBEC family of single-stranded deaminases (APOBEC3B) to generate mice with enhanced mutational burden (KPA mice) (208). Increased mutations produce neoantigens, enabling the immune system to recognize the tumor. Consistent with Joshi et al., there was no observed ectopic immune cell accumulation in KP, nor in the KPA mice, at least in proximity to the tumor. To assess whether immune cells altered tumor growth, they created a KPA mouse line on a Rag1-/- background (KPAR mice), but the tumor load remained unchanged and even led to a lethal tumor load in about 14 weeks without activating an anti-tumor response (208). The authors attributed the absent immunogenicity to the subclonal mutations caused by ectopic APOBEC expression. Therefore, they subsequently generated single-cell cloned lines from KPAR tumors and found that upon i.v. injection KPAR tumors developed in the lungs of C57BL/6 mice. Most importantly, the orthotopic tumors induced an anti-tumor response, including CD4+ and CD8+ T cells, as well as NK-cell infiltration. Interestingly, the authors observed the expression of a viral glycoprotein from the murine leukemia retrovirus in the KPAR cells and concluded that endogenous retroviral antigens can trigger effective CD8+ T cell responses. Finally, the tumor growth of KPAR tumors was reduced upon ICB. In a recently published study, the KPAR orthotopic model was used to demonstrate B cell accumulation near the tumor (209). Ng et al. detected mature TLS containing GCs and serum antibodies against KPAR cells expressing endogenous retrovirus envelope glycoproteins. Furthermore, the titer against the virus antigens increased upon ICB with anti-PD-L1 antibodies, shedding light on the dependence of effective anti-tumor B cell responses on viral antigens. The expression of retroviral antigen was also detected in LUAD patients as a prerequisite for response to ICB therapy. In addition, they demonstrated the curative effect of CXCL13 therapy in combination with ICB to enhance anti-tumor immunity in the KPAR orthotopic model. In summary, GEMMs, which contain multiple mutations similar to human LUAD tumors, have been highly useful in investigating the effect of immunotherapy concepts such as ICB in combination with soluble factors that enhance TLS formation. In addition to GEMMs, the i.v. injection of B16 melanoma cells is widely used as a model for melanoma metastasis in the lung, as most, if not all, injected tumor cells accumulate in the lung and induce the formation of lymph node-like structures that include HEVs (215). The B16 melanoma cell line originated from a spontaneous tumor in a C57BL/6J mouse (216). Nevertheless, this transplantation model may not actually be a model for LUAD or lung metastasis, as all B16 tumors should be considered as primary tumors that do not accurately mimic human LUAD (217).

## Pancreatic ductal adenocarcinoma TLS models

To model PDAC, either GEMMs or orthotopic transplantation models have been employed. Much like the KP model in the lung, a mouse strain expressing the mutated Kras G12D in pancreatic ductal cells (LSL-KrasG12D/+;Pdx-1-Cre; KC mice) was developed, resulting in a low tumor load after 5 months (218). Subsequently, after the additional depletion of p53 (LSL-KrasG12D/+;LSL-Trp53R172H/+;Pdx-1-Cre; KPC mice), KPC mice developed tumor-associated TLS, including GC B cells (219, 220). Interestingly, Spear et al. did not observe TLS formation in an orthotopic PDAC model where a KPC-derived cell line from liver metastasis was injected into the pancreas (220). This absence was likely due to the highly proliferative nature of this model, resulting in tumor formation within two weeks. Tseng et al. implanted KPC-tumor cells into the pancreas of syngeneic mice without detecting prominent TLS (221). However, transplantation of KPC-tumor cells into Rag-deficient recipients resulted in lower survival compared to immunocompetent mice, suggesting anti-tumor responses by B and T cells primed in SLOs. The KP model, involving spontaneous tumor formation, was combined with DNA vaccination against α-enolase, whose expression is increased in PDAC cells. This vaccination induced the formation of GC B cells and recruitment of T cells into the tumor, thereby fostering TLS formation (222). Additionally, in a model of s.c. transplantation of a human PDAC cell line, coupled with intratumoral injection of CCL21, Turnquist et al. observed increased accumulation of T cells, DCs, and NK cells, forming a pre-TLS structure. This indicated the importance of chemokine guided migration into the tumor, which may show potential for immunotherapy (210). Of note, the overexpression of lymphotoxin in the pancreas during steady-state successfully induced the formation of TLS, suggesting potential for including this construct in KP and KPC models in the future (223). Using an orthotopic transplantation of KPC tumor cells, the i.v. treatment with nanoparticles containing the antifibrotic compound α-mangostin and a plasmid encoding LIGHT resulted in reduced tumor growth. This was accompanied by reduced activated fibroblast numbers, decreased collagen deposition, normalized tumor vasculature, and most importantly, the induction of organized TLS in the tumor (224). These results highlight the intriguing role of extracellular matrix organization in TLS formation. In summary, KP and KPC mice are valuable tools for studying TLS formation, especially concerning DNA vaccines and chemokine therapy. Future studies will reveal if these models can be utilized to study the interplay between TLS and ICB.

## Hepatocellular carcinoma models show immunosuppressive features of TLS

The tumorigenesis of HCC was investigated in GEMM by Finkin et al. (93). The authors developed two models of inflammation-driven HCC in mice with an overactive NF-κB signaling pathway, a typical feature of human HCC. IKKβ(EE)Hep mice display a hepatocyte-targeted, constitutively active NF-κB pathway after breeding them with a suitable deleter strain (Albumin-Cre mice). Within 7 months, typical hallmarks of liver inflammation, including an accumulation of macrophages, liver damage, hepatocyte proliferation, and structured TLS with B and T cells as well as HEVs, were detected. After 20 months, all mice developed HCC, indicating that TLS were formed prior to tumorigenesis. Next, the authors generated Alb-IKKβ(EE) mice with constitutively active NF-κB signaling in hepatocytes without the requirement of a Cre recombinase. These mice showed accelerated tumor and TLS growth within 9 months. Interestingly, on a Rag1-/- immunodeficient background, Alb-IKKβ(EE) mice showed a drastically reduced tumor burden, illustrating the pro-tumorigenic effect of the adaptive immune system. In fact, TLS, located in proximity to the tumor but not within the tumor, served as a niche for forming HCC progenitor cells that later egressed and developed into HCC. In summary, the overexpression of the NF-κB signaling pathway in a spontaneous model of HCC mimicked human disease and was suitable for analyzing new concepts of immunotherapy, including interference with TLS formation (225). Future studies are required to develop a relevant model for intratumoral TLS formation that does not show the tumor-supportive features discussed above.

## TLS formation in colorectal carcinoma models

To investigate spontaneous tumorigenesis in the intestine, the APCmin model carrying a mutant allele of the APC locus, similar to the mutation in humans, is frequently used, and the accumulation of immune cells in proximity to adenomas and/or tumors is described (our own observations and (226)). In addition, the (repetitive) i.p. injection of the carcinogen azoxymethane (AOM) can be used to induce colon tumorigenesis in mice harboring different alterations in intestinal epithelial cells, such as the deletion of p53 (227). However, a targeted analysis of TLS formation in such models is currently lacking.

**Figure 2:**
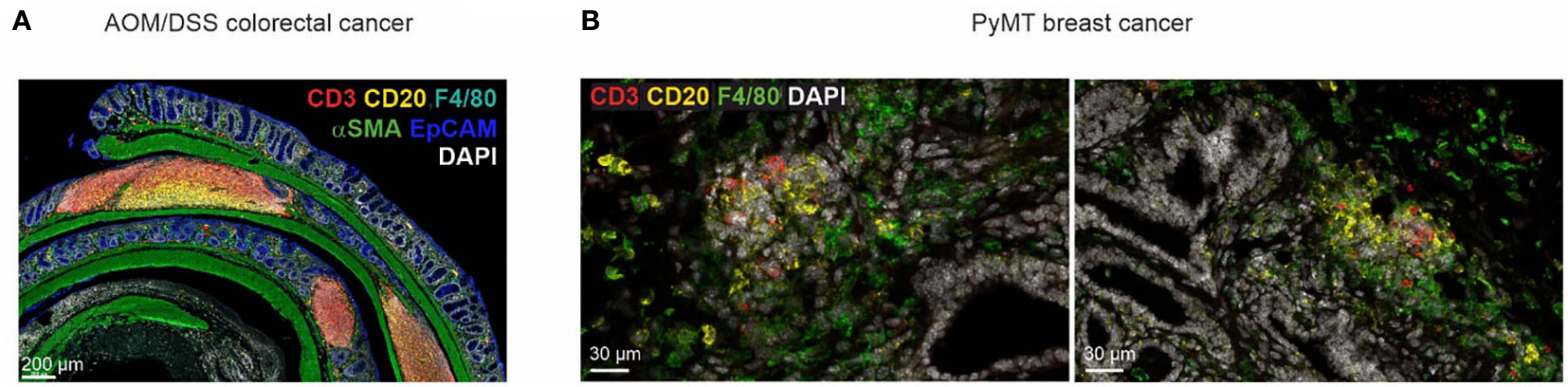
TLS heterogeneity in cancer mouse models. **(A)** TLS in different maturation stages in a tumor-bearing mouse in the AOM/DSS model of CRC. **(B)** Early lymphocyte aggregates in the PyMT mouse model of invasive mammary carcinoma.

The most common model used to investigate inflammation-driven tumor formation that mimics tumorigenesis in patients with inflammatory bowel diseases is the AOM/DSS model, where the animals receive one injection of AOM and three repetitive cycles of dextran sodium sulfate salt (DSS) in the drinking water (228). Our own observations (**Figure 2A**) clearly show the formation of organized TLS in the AOM/DSS model, but information how TLS formation occurs and whether it can be manipulated, e.g., by ICB in this model, is lacking. Interestingly, a recent study showed that the intestinal microbiota plays an important role during tumorigenesis and can also affect TLS formation in the AOM/DSS model (229). The authors detected increased anti-tumor immunity and reduced tumor growth upon the introduction of *Helicobacter hepaticus (Hhep)* into C57BL/6 animals in the AOM/DSS model. In fact, *Hhep* induced the expansion of T follicular helper cells, leading to the formation of organized TLS in proximity to the tumor. These results indicate that the microbiota has the power to induce the formation of TLS in an inflammatory environment. Importantly, the formation of TLS appeared to be rather independent of tumorigenesis *per se* but was still useful in inducing an anti-tumor immune reaction.

In addition to the models indicated above, the orthotopic injection or enema of genetically engineered tumor organoids is a novel approach to mimic the mutational cascade in human CRC. These models overcome the limitation of AOM and AOM/DSS tumors, which do not induce metastases (230). Again, TLS formation in orthotopic colon tumors has not been described so far. However, patient-derived organoids (PDO) transplanted into the caecum of humanized mice generated tumors that formed metastases in the liver and peritoneum, but only the growth of the primary tumor and liver metastases were diminished upon ICB treatment (anti-CTLA4, anti-PD-1), a phenomenon also observed in CRC patients (231). The authors detected structured TLS in the primary tumor and the liver metastases but not in the peritoneum. The TLS contained T cells and B cells, showing an IFN-γ signature and CXCL13 expression. Therefore, alterations in tumor growth upon ICB were correlated with the presence of TLS. In summary, although many GEMMs and GEMM-derived organoids are available to model CRC, they are presently underused to investigate TLS during tumorigenesis *in vivo*.

## Evidence for early TLS formation in breast cancer models

For investigating the development of breast cancer an autochthonous mouse was developed in 1992 by fusing the mouse mammary tumor virus (MMTV) long terminal repeat promotor with the polyomavirus middle T antigen (PyMT), resulting in tumor formation in mammary glands and lung metastasis (232). While PyMT is not a human oncogene, MMTV-PyMT mice develop similar features, especially compared to end-stage human breast tumors: such as the loss of estrogen receptor and progesterone receptor expression, as well as overexpression of ErbB2 and Cyclin D1 (233). Our own observations (**Figure 2B**) suggest the formation B and T cell aggregates at the tumor margins but not within tumors. A detailed investigation of structured TLS formation in this model is currently lacking. Interestingly, a combined anti-angiogenic and anti-PD-L1 therapy approach in PyMT mice induced the formation of intratumoral HEVs, which might serve as a prerequisite for TLS formation (234). Furthermore, a more recent detailed analysis of this model with anti-angiogenic and anti-PD-L1 treatment showed the transition from tumor endothelial cells into HEVs based on LT-β receptor signaling by NK and CD8 T cells, thus promoting the expansion of anti-tumor effector T cells (75). Future investigations are essential to confirm the formation of TLS in the PyMT model, as well as in other breast cancer models, such as the inducible expression of viral antigens in mammary epithelial cells (235), aiming to mimic human breast cancer. The PyMT model produces immunologically rather cold tumors that do not respond well to ICB (236, 237), despite frequent mutations in this model (233). Therefore, strategies to overcome immunosuppressive mechanisms in this model are likely necessary to enable the investigation of TLS formation.

## TLS formation in models of melanoma

An autochthonous model for melanoma was established in 2009 by inducing the expression of a constitutively active BRAF mutation at position 600 (BRAF^V600E/+^) under the control of the inducible keratinocyte-specific Cre recombinase Tyr::CreERT2 (238). These animals showed highly pigmented lesions, but did not develop malignant melanoma. Therefore, the authors generated a new mouse line by introducing the tumor suppressor Pten with floxed Exon 4 and 5 (BRAF^V600E/+^ PTEN-/- Tyr::CreERT2). Upon repetitive topic application of Tamoxifen, these mice developed melanoma with metastases in lymph nodes and lungs (238). In these melanomas, tumor infiltrating lymphocytes, including CD4+ T cells, CD8+ T cells, Tregs, and DCs, were found, but organized lymphoid structures *in situ* were not investigated (239). Another group observed the early influx of Tregs at the onset of melanoma development, followed by CD8+ T cells (240). Upon the depletion of Tregs, they observed an increased activity and clustering of tumor-infiltrating lymphocytes. This illustrates that Tregs can prevent the development of anti-tumor immunity and the formation of TLS. For the development of differentiated TLS, the formation of PNAd+ HEVs is crucial as they enable the influx of immune cells directly into the TLS. Peske et al. did not detect HEV formation in the BRAF^V600E/+^ PTEN-/- Tyr::CreERT2 mouse model (76). In summary, this autochthonous melanoma model in its current form is not suitable for a comprehensive analysis of TLS. Integrating a strategy for inducible depletion of Tregs might optimize the model towards this goal.

## Using transplantation models to study TLS formation

The transplantation of tumor cells i.p, i.v., or s.c. into immunocompetent as well as in immune-deficient animals has proven to be a useful method for investigating anti-tumor immune mechanisms, including ICB (241), and the formation of TLS. Various cell lines have been used, depending on the specific research question. The most frequently used cancer cell line is the B16 melanoma cell line that was isolated from a spontaneous melanoma in a C57BL/6 mouse (216). B16 cells produce melanin, making them easy to track in the host. Numerous sublines were created expressing artificial antigens or tumor-associated antigens. In general, transplantation of tumor cells offers several advantages, as tumors develop rapidly within two weeks and can be transfected to induce model antigen expression or harbor mutations that are similar to human cancers. The choice of the recipient mouse line allows the investigation of the role of different immune cells. For example, Rag1-/- mice lack B and T cells, whereas µMT mice lack only B cells. Furthermore, the choice of immunocompetent mouse lines harboring a specific knockout, such as deficiency to produce lymphotoxin, can help analyze the role of individual factors during TLS formation (211). It may be surprising that such rapid models are suitable to study TLS formation. However, rapid development of TLS in mice triggered by chronic inflammation that is not related to tumor formation is well established (31, 32). Often, s.c. injection of tumor cells into mice may trigger acute inflammatory reactions rather than mirror the tumor immune co-evolution seen in humans. Therefore, it remains to be determined if TLS emerging under such conditions accurately reflect the situation in human tumors. Along these lines, the method of application appears to be crucial in investigating TLS formation in transplantation models. Two studies reported TLS formation upon B16-OVA melanoma cell transplantation i.p., but not s.c (76, 242). It was further described that fibroblasts in B16-OVA i.p. and s.c. tumors showed differential expression of adhesion molecules. When Icam1+Vcam+ fibroblasts were transplanted together with B16-OVA cells s.c., TLS formed, but not when Icam1+Vcam- fibroblasts were used (54). Most importantly, organized TLS were only detected after ICB, confirming the importance of reducing anti-inflammatory pathways to induce effective anti-tumor immunity (54). The importance of CCL21 for TLS formation was demonstrated in the B16 melanoma model upon s.c. transplantation with different sublines over- or under-expressing CCL21 (243). B16 tumors with high CCL21 expression induced TLS formation but a successful anti-tumor response was prevented due to the infiltration of suppressive immune cells such as Tregs. This illustrates the complex interplay of chemokines required for the formation of immunogenic TLS. In another study, B16 cells were modified to express the tumor-specific sphingolipid GD2 and injected i.v. into syngenic mice, resulting in the formation of pulmonary tumors (215). The authors created a fusion protein of a chimeric anti-GD2 antibody fused to lymphotoxin and demonstrated its effectiveness in reducing the growth of lung tumors by inducing peritumoral TLS, including T cells, B cells, and HEVs. Interestingly, tumor-specific T cells were detected, indicating the effective activation of naïve T cells within the TLS. The potency of lymphotoxin was further highlighted by transplantation of B16 cells expressing GD2 into lymphotoxin-deficient mice (211). Despite the absence of secondary lymphoid organs, TLS were formed upon treatment with the fusion protein.

In a different approach, genetic manipulation of DCs to produce Tbet (DC.Tbet) was reported as a useful tool to induce TLS (63). The authors induced tumors by s.c transplantation of MCA205 sarcoma cells. After seven days, they performed a therapeutic injection of DC.Tbet cells i.t. and observed reduced tumor growth and the development of anti-tumor immunity in association with TLS formation, including the accumulation of T cells, B cells, NK cells, DCs, and PNAd+ HEVs. Interestingly, DC.Tbet cells already secreted CCL19, CCL21, LIGHT/TNFSF14, and lymphotoxin, thereby inducing TLS formation even in lymphotoxin-deficient mice transplanted with MCA205 sarcoma cells and treated with DC.Tbet cells. DC.Tbet cells further produced high levels of IL36γ, and upon transplantation of MC38 colorectal cancer cells into IL36 receptor-deficient mice, the formation of TLS was impaired. These studies indicated a novel role for IL36 in anti-tumor immunity during TLS formation.

We do not offer a complete list of all transplantable tumor models that develop TLS, but rather aim at highlighting models that are particularly useful for investigating TLS formation. While many studies describe the induction of successful anti-tumor immune responses without focusing on TLS formation (244, 245), the diversity of tumor cells and recipient mice, particularly when considering genetic modification of one or both, makes s.c. and i.p. transplantation models valuable tools to investigate at least early mechanisms of TLS formation.

## Conclusions

The data summarized above establishes the prognostic relevance of TLS for cancer patients, while outlining the challenges that lie ahead when considering TLS formation as a reliable prognostic and therapeutic goal. While the suitability of TLS as biomarkers in different tumor entities is solidifying, the signals to initiate, sustain, but also prevent TLS formation, and the cellular interactions within TLS, are poorly understood. Such knowledge would be necessary to envision targeted induction of TLS in cancer and prevent their undesired formation in other contexts. Along these lines, an important aspect to consider is the potential auto-immune reactions that may be triggered when TLS are therapeutically induced. While the antigen-dependence of TLS formation may be a limiting factor for such side-effects, recent studies have indicated that TLS may also form due to chronic inflammation and aging without the requirement of specific antigens (32). The requirement of a sufficient degree of ongoing inflammation for TLS formation not only emerges from cancer mouse models, such as the PyMT model discussed earlier, but also from clinical observations. For instance, tobacco exposure, which triggers inflammatory reactions, has been connected to increased TLS abundance and CCL21 in lung adenocarcinoma patients, correlating with the response to immunotherapy (246). Moreover, cancer patients undergoing corticosteroid treatment exhibited impaired TLS maturation or formation (190, 247). Other inflammatory triggers, such as dying cells or related DAMPs commonly found in tumors and induced in response to tumor therapy, may also affect TLS development (69).

**Figure 3:**
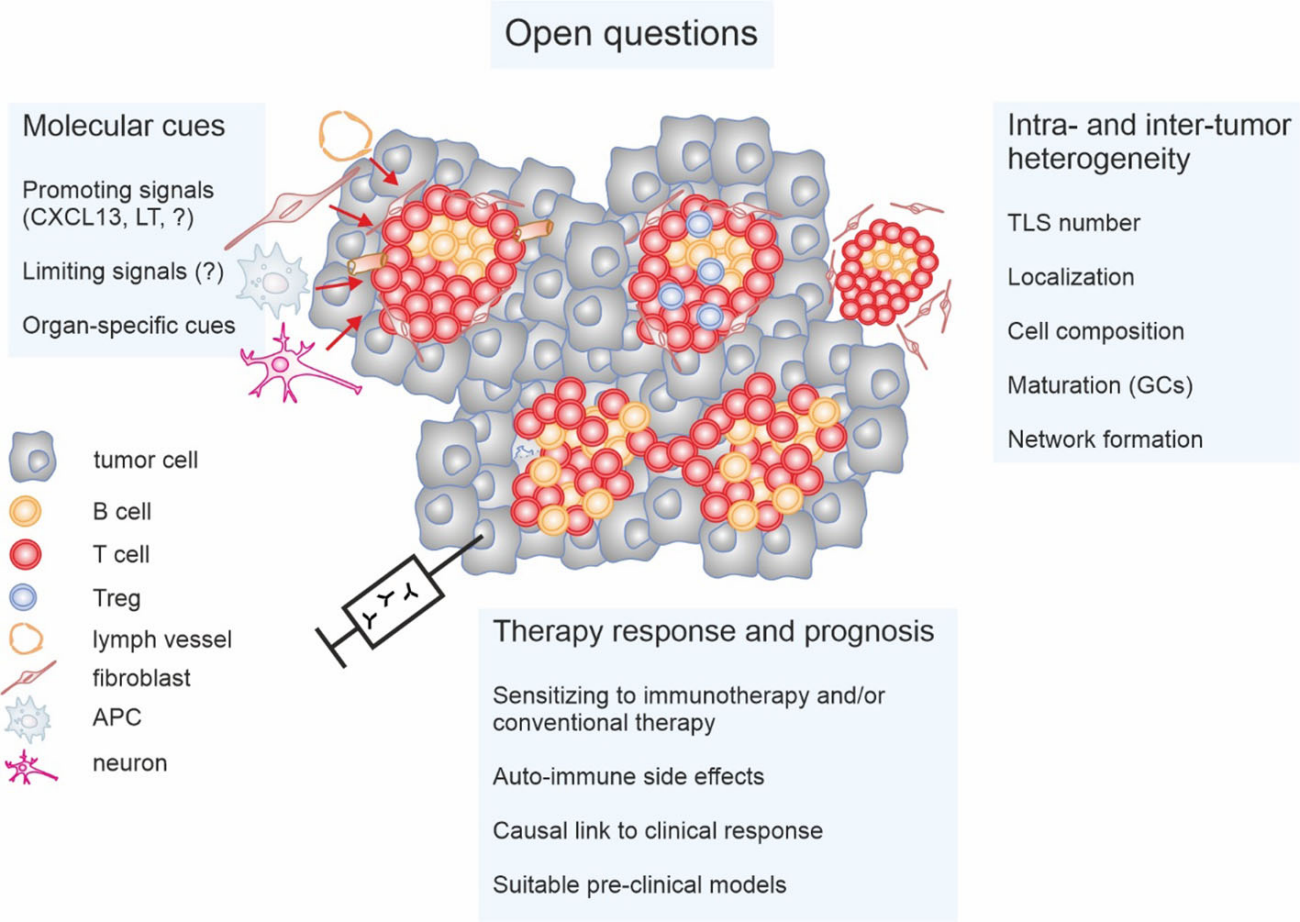
Open questions concerning tertiary lymphoid structures (TLS) in cancer. The impact of the intra- and inter-tumor heterogeneity of TLS on anti-tumor immunity and patient prognosis is not fully understood. Important parameters appear to be not only the number of TLS and their localization. Cellular composition such as the relative abundance of regulatory T cells (Tregs) and maturation status such as the presence or absence of germinal centers (GCs) as well as interconnectivity throughout tumors may play a role. Moreover, signals that on the one hand induce and on the other restrict TLS formation, and the cells providing these signals, need to be identified and/or better characterized. This is also relevant on the context of organ specificity. Finally, the fundamental question if TLS are causally involved in anti-tumor immunity or represent a bystander phenomenon is still unanswered. This is only relevant to be able to understand the impact of TLS on anti-cancer therapy responses, and if induction of TLS formation would induce unacceptable auto-immune side-effects. Research towards answering these open questions will require suitable pre-clinical models.

There are critical questions in the TLS field that we feel need specific attention (**Figure 3**). Particularly TLS heterogeneity concerning maturation state, location, interconnectivity, and cellular composition, both globally and in discrete tumor niches at primary and metastatic sites, needs consideration. The impact of these parameters on anti-tumor immunity and disease progression requires further clinical and pre-clinical investigation. Moreover, standardized, clinically applicable methods of TLS detection need to be established. Importantly, it is not entirely clear if TLS are simply indicative of the local immune response in a tumor or if they represent relevant anti- or pro-tumor entities by themselves. Understanding this issue is vital not only for targeted TLS induction but may also be of interest when aiming at avoiding auto-inflammatory side-effects of immune activation against tumors. Suppressing TLS may, in some cases, reduce auto-immune side-effects rather than affecting anti-tumor immune responses.

Addressing these questions requires longitudinal and spatial analyses to compare intra- and extra-tumoral immune responses, preferably in suitable mouse models that closely replicate tumor development in humans. Such experiments would yield strategies to selectively induce or deplete TLS without hampering local extra-TLS immune responses as well as SLO-dependent adaptive immunity. Another pressing question is identifying the signals and cellular composition that render TLS activating versus suppressive. Understanding the signals that induce or suppress Treg formation/activity in TLS and targeting these mechanisms could potentially revert suppressive TLS into immune-stimulatory powerhouses. Phenotypes and functional properties of suppressive Tregs in TLS and their putative association with TLS maturation require further investigation.

Finally, not all tumors seem permissive for TLS formation, suggesting that homeostatic signals may limit TLS induction even in the presence of inflammatory stimuli. Cytokine receptor antagonists, as shown for IL-36RA (63), or cytokine and chemokine decoy receptors (248) might be potential candidates. Blocking these mediators and receptors may, thus, trigger TLS formation even in non-permissive environments. However, the potential risk of auto-inflammatory side-effects also requires investigation. To this end, optimal tumor (mouse) models to study TLS formation that mimic both, the time scale and cellular complexity of tumor formation in humans need to be developed and cross-validated. Such studies will reveal the true benefit of interfering with TLS formation in cancer patients.

## Author contributions

XY: Data curation, Investigation, Writing – original draft, Writing – review & editing. KK: Funding acquisition, Writing – original draft, Writing – review & editing. AW: Data curation, Funding acquisition, Supervision, Visualization, Writing – original draft, Writing – review & editing.
